# Supplementary material for: Associations Between Problematic QQ Use and Mental Health Among Chinese Children and Adolescents: A Latent Class Analysis
Source: Brain Sci. 2025 Oct 27;15(11):1148. doi: 10.3390/brainsci15111148 (PMC12650318; doi:10.3390/brainsci15111148)
Supplement: Supplementary file 1 [file brainsci-15-01148-s001.zip › brainsci-3934060-supplementary.pdf]

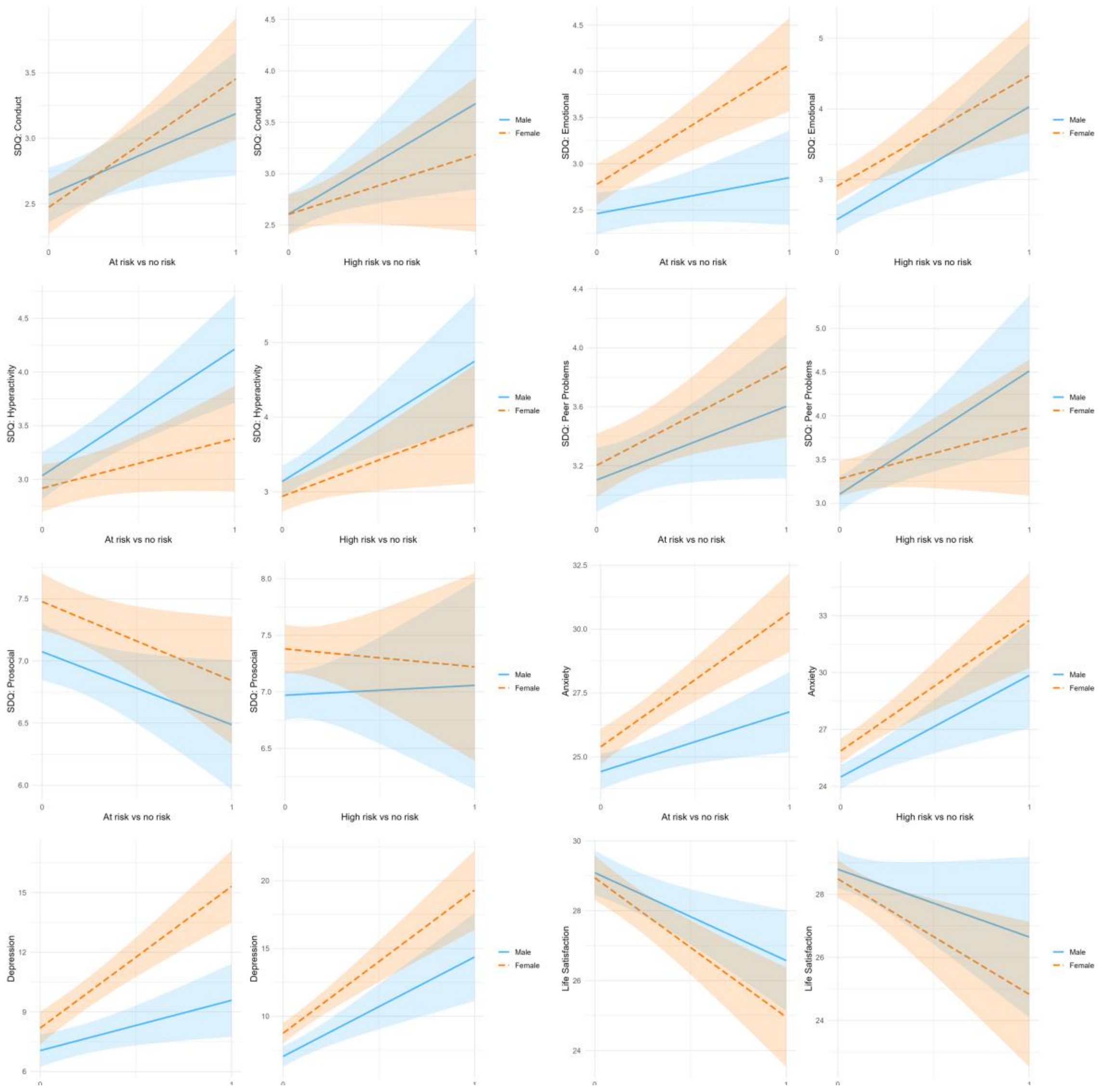

Note. SDQ = Strengths and Difficulties Questionnaire.

Figure S1. Moderating Effect of Sex on the Association between Latent Group of QQ users and Mental Health.
